# The AI inversion model: a linear negative-constraint framework for auditable alignment in medical decision-making

**DOI:** 10.1186/s12910-026-01488-2

**Published:** 2026-05-27

**Authors:** Eyal Cohen, Rachel Nissanholtz-Gannot, Yehuda Adler

**Affiliations:** Faculty of Health Sciences, Ariel University, Ariel, 40700 Israel

**Keywords:** AI Alignment, Algorithmic Bias, Medical Ethics, Negative Constraints, Genesis Anchor System, Vector Kinematics, Autonomous Calibration, Proof-of-Concept (PoC), Clinical Decision Support Systems (CDSS)

## Abstract

The integration of artificial intelligence (AI) into healthcare systems is increasingly hindered by the AI alignment problem. In high-stakes domains such as clinical triage, algorithms frequently reflect and amplify systemic biases. Current alignment methodologies, including Reinforcement Learning from Human Feedback (RLHF), attempt to encode subjective human morality through opaque architectural pipelines, which can exacerbate the “black box” dilemma.

To address this, this paper proposes the “AI Inversion Model”, a theoretical Proof-of-Concept (PoC) framework utilizing inference-time negative constraints. Rather than attempting to compute elusive positive virtues, the model draws clinical inspiration from the psychopathic spectrum - specifically extreme utilitarianism - to define strict “anti-moral” boundaries. These boundaries are operationalized through a “Genesis” anchor system, which autonomously and recursively calibrates negative anchors by synthesizing supreme legal precedents and ethical literature.

Beyond static distance, the model evaluates the mathematical kinematics of AI outputs - including vector angles and directional trajectories - to quantify algorithmic “Good Faith”. A step-by-step trace of complex surgical simulations illustrates how the filter identifies utilitarian biases and enforces a human-in-the-loop protocol when variance exceeds a heuristic 10% threshold. While not a definitive resolution to the black-box problem, this model offers a targeted, auditable baseline of “functional compassion” to mitigate systemic bias in medical AI governance.

**Supplementary Information:**

The online version contains supplementary material available at 10.1186/s12910-026-01488-2.

## Introduction & background

### The alignment problem in medical artificial intelligence

The accelerated integration of artificial intelligence (AI) into global healthcare systems offers unprecedented potential to improve diagnostic accuracy, streamline clinical triage, and optimize data-driven resource allocation [1]. However, this technological advancement is accompanied by a severe ethical challenge widely recognized in the literature as the “AI alignment problem”-the inherent difficulty of ensuring that autonomous systems operate consistently with normative human values, particularly in complex, high-stakes environments such as medicine [2, 3].

In the domain of medical decision-making, algorithms directly shape human lives and outcomes. Yet these models frequently reflect, replicate, and even amplify systemic biases embedded within their historical training data. Consequently, AI systems risk producing decisions that systematically discriminate against protected classes, including disparities based on age, gender, race, or socioeconomic status [4, 5].

While many algorithms are fundamentally optimized to maximize an objective function of clinical or economic efficiency (utilitarianism), this uncompromising pursuit of utility can routinely override fundamental principles of equity, distributive justice, and medical beneficence [3]. The result is a paradigm where decisions that are statistically “correct” may manifest as profoundly apathetic or cruel toward human suffering.

### The “black box” dilemma and the limitations of RLHF

To mitigate algorithmic bias and address safety risks, the industry and academia have widely adopted alignment methodologies such as Reinforcement Learning from Human Feedback (RLHF) [6]. Although RLHF has proven effective in refining the behaviour of large language models (LLMs) and preventing overtly offensive outputs [7], it suffers from a profound structural limitation known as the “black box” dilemma [8].

When a Clinical Decision Support System (CDSS) based on such a model outputs a medical recommendation that discriminates against a vulnerable or elderly patient, the attending physician cannot trace the computational pathway that led to that specific decision [9]. This architectural opacity fundamentally precludes the generation of a coherent audit log. In medicine - a field predicated on accountability, explainability, and trust - the inability to explain why a specific patient was denied intensive care renders the technology ethically and administratively untenable [8, 10].

#### The central thesis: the AI inversion model (proof-of-concept) 

Considering the limitations inherent in RLHF and the computational encoding of positive morality, this manuscript proposes a theoretical Proof-of-Concept (PoC) framework: The AI Inversion Model. Rather than attempting to teach the machine what constitutes a “good” moral action, the framework focuses on the explicit definition of what constitutes an “anti-moral” action, subsequently blocking it via inference-time negative constraints.

To anchor these boundaries in a framework less susceptible to subjective cultural interpretation, the proposed model draws clinical inspiration from psychiatry, specifically the psychopathic and sociopathic spectrums [11]. In psychiatric literature, psychopathic traits are characterized by specific cognitive and emotional deficits: a profound apathy toward the distress of others, cold utilitarianism, and a propensity to manipulate rules to achieve a target outcome [11].

To operationalize these boundaries, the system employs the “Genesis” anchoring method. The negative pole (representing “Absolute Evil” or psychopathic utilitarianism) and the positive pole (representing “Absolute Necessity”) are not manually hardcoded heuristics. Instead, the Genesis system is designed to perform autonomous calibrations by recursively synthesizing dynamic streams of supreme legal jurisprudence, statutory norms, and high-impact medical ethics literature. By establishing these data-driven extremes, the system constructs an auditable baseline.

Furthermore, to combat adversarial inputs and algorithmic manipulation, this mathematical framework evaluates more than just static semantic proximity. It incorporates vector kinematics-analysing the angle, velocity, and directional trajectory of the AI’s semantic shifts-to quantify the algorithm’s operational “Good Faith”. If the system’s proposed action deviates from the optimal normative center and breaches a strict 10% distance threshold (moving too close to the negative anchor), the action is automatically flagged and blocked.

Through the implementation of these negative constraints, the PoC model does not claim to produce software with superior morality. Rather, it establishes a reliable, computationally tractable baseline of “functional compassion” that is fully quantifiable and technically auditable to mitigate systemic bias in modern healthcare.

## Conceptual framework & literature review

### Situating the model within contemporary AI governance

Current approaches to mitigating algorithmic bias in healthcare primarily fall into two paradigms: Explainable AI (XAI) and algorithmic fairness models [4]. While both represent significant advancements, they exhibit structural limitations when applied to the acute complexities of clinical triage. XAI methodologies predominantly function as post-hoc diagnostic tools; they may explain a biased recommendation after the fact, but they do not inherently prevent it during inference [4]. Conversely, rigid algorithmic fairness models can be clinically detrimental, as medical triage inherently requires justified, evidence-based prioritization [3, 4].

The AI Inversion Model (PoC) introduces a distinct third paradigm: active, inference-time negative constraints. Rather than forcing statistical parity, the model acts as an auditable guardrail that continuously evaluates the algorithmic output against “anti-moral” thresholds. This is achieved through the “Genesis” anchoring system, which ensures that the defining poles of ethical behaviour are not static or subjective, but are autonomously derived from authoritative legal and ethical corpora.

### The philosophy of negative constraints: from clinical markers to genesis 

To establish these “anti-moral” thresholds, the Inversion Model utilizes clinical psychiatry—specifically the diagnostic constructs of psychopathy and sociopathy—as an epistemological anchor [11]. In the context of algorithmic governance, a machine learning model optimizing for efficiency without a programmed constraint to weigh human suffering acts in a mathematically analogous manner to psychopathic apathy: it is ruthlessly efficient, yet entirely indifferent to clinical distress [11].

By defining these negative boundaries, the system is not tasked with the computationally intractable goal of calculating “perfect virtue”. Instead, it is programmed to recognize and invert the “most apathetic” action. This process is governed by the Genesis mechanism, which recursively synthesizes supreme court rulings and high-impact bioethics literature to ensure the negative anchor (Absolute Evil) accurately reflects the highest normative standards of medical law and ethics.

### Operationalizing indifference: the transition to vector kinematics

To transition from philosophy to a computationally tractable model, clinical markers of apathy must be translated into measurable variables. The Inversion Model deconstructs any medical recommendation into dynamic weights: Systemic Utility $\left({W}_{efficiency}\right)$ , Patient Distress $\left({W}_{distress}\right)$ , and Protected Attributes $\left({W}_{bias}\right)$ .

In this PoC framework, “algorithmic apathy” occurs when the model’s output indicates that has completely diluted . Mathematically, this is evaluated by measuring the similarity of the decision vector to the established Genesis poles.

Crucially, as will be detailed in the following section, the model extends beyond static distance to incorporate Vector Kinematics - monitoring the angle and velocity of the semantic transformation to detect bad - faith manipulations or “gaming” of the ethical filter. This provides a transparent, quantifiable ratio of indifference that can be actively audited without accessing the underlying neural weights of the AI.

## Methodology & the linear model

### Translational architecture: from semantics to vectors

To operationalize the theoretical boundaries of the AI Inversion Model, the framework utilizes Natural Language Processing (NLP) embeddings and linear algebra to evaluate the semantic properties of an AI-generated medical decision. The model deconstructs clinical events into recursive sub-events, mapping each into a multidimensional vector space where ethical values are represented as coordinates between 0 and 1.

Central to this architecture is the “Genesis” Anchor System. Unlike static models that rely on manual weight adjustments, Genesis ensures absolute autonomy by performing daily calibrations against external normative corpora. The system autonomously ingests supreme legal precedents, statutory healthcare norms, and high-impact bioethics literature to dynamically establish the two reference poles:

I. *The Negative Pole* (“*Absolute Evil*”): Represents the “nadir” of clinical apathy - extreme utilitarianism and bad-faith manipulation derived from synthesized jurisprudence and psychiatric markers [11].
II. *The Positive Pole* (“*Absolute Necessity*”): Represents optimized medical beneficence and justified triage, serving as the normative center $\:\left(0.5\right)$

### Mathematical formulations and vector kinematics

The evaluation of the AI’s proposed decision is executed through a deterministic sequence of linear equations. To ensure the system remains unassisted by human intervention and resistant to adversarial “gaming”, the model incorporates Vector Kinematics.

#### Vector normalization

$$\:v=\frac{v}{\parallel{v}\parallel}$$

##### Philosophy

Converts text embeddings into unit vectors, ensuring comparisons are based purely on semantic direction (intent) rather than text length or verbosity.

#### Cosine distance and similarity

$$\:{D}_{cos}\left(A,B\right)=1-\frac{A\cdot\:B}{\parallel{A}\parallel\parallel{B}\parallel}$$

$$\:{S}_{good}=1.0-{D}_{cos}\left(case,pos\right)$$

$$\:{S}_{bad}=1.0-{D}_{cos}$$

##### Philosophy

Quantifies the geometric divergence between the AI’s logic and the Genesis poles.

#### Kinematic evaluation (good faith metric)

Beyond static distance, the system measures the velocity$\:\left({V}\right)$ and angle $\:\left({\uptheta\:}\right)$ of the semantic shift during the decision-generation process.  

I. *Directional Trajectory*: If the vector angle deviates toward the negative anchor while maintaining high “semantic velocity,” the system identifies a lack of Good Faith (Reasonableness).
II. *Recursive P-Score (Dilution Core):*

$$\:P=\frac{{S}_{bad}}{{S}_{good}+{S}_{bad}}$$

##### Philosophy

Translates relative apathy into a mathematical dilution. It measures the exact degree to which biased utility overrides clinical distress.

**Computational implementation note**

The deterministic mathematical formulations described above, including the autonomous anchor calibration, kinematic drift evaluation, and the P-score dilution algorithm, have been computationally operationalized. To maintain the narrative flow of the manuscript, the complete Python codebase demonstrating this inference-time Proof-of-Concept (PoC) is provided in Appendix A (Supplementary Material) at the end of this manuscript.

### Governance: daily autonomous calibration

The AI Inversion Model operates as an independent, inference-time guardrail. To eliminate human-in-the-loop calibration bias, the Genesis system maintains the integrity of the poles through a rigid 90% distance constraint.

The system autonomously updates its semantic anchors every 24 h by scanning the latest medical-legal databases. This ensures that the “Absolute Evil” pole is always anchored in the most current societal and legal definitions of clinical negligence and apathy. By maintaining a mandatory 10% safety buffer, the model guarantees that any decision vector moving within the proximity of the negative anchor is immediately blocked.

### Verdict thresholds (hard constraints) and justification

- ➢ If *P* < 0.45: APPROVED (Safe Baseline)
- ➢ If *P* > 0.55: REJECTED (Human-in-the-Loop Triggered)

Threshold Status and Justification: It is critical to state explicitly that the specific values of 0.45 and 0.55 utilized in this framework are heuristic placeholders intended strictly for this theoretical Proof-of-Concept (PoC) demonstration. They have not yet been empirically derived or validated through large-scale clinical datasets. In a fully operationalized clinical environment, these thresholds would not be static or universally applied. Rather, they would require precise parameterization by institutional ethics committees based on department-specific risk tolerances (e.g., intensive care versus elective surgery) and continuous sensitivity testing to balance false-positive and false-negative rates.

Philosophy: Within the context of this PoC, these provisional thresholds represent the active negative constraints. A breach of the 0.55 threshold theoretically indicates that the output is saturated with utilitarian bias that overrides functional compassion. At this junction, the system generates a transparent audit log and halts the process for human oversight.

## Clinical simulation – the surgical decision space

### The complexity of intraoperative triage and the anesthetized patient

Intraoperative environments represent a high-stakes, dynamic “decision space” where the patient - rendered unconscious and surgically vulnerable - is entirely dependent on the ethical integrity of the clinical team and their supporting algorithms [1, 2]. Unlike static triage, surgical decisions are composed of recursive micro-events, each carrying the risk of “algorithmic apathy”. Medical negligence in these contexts often manifests not as an overt error, but as subtle shifts toward efficiency-driven shortcuts that breach the established standard of care [3].

### System architecture: offline autonomy and internalized genesis anchors

A critical feature of the AI Inversion Model is its operational self-sufficiency. To ensure maximum data privacy and eliminate reliance on external connectivity within the sterile operating environment, the Genesis Anchor System operates using an extensive, pre-calibrated internal database [2]. This “ethical memory” contains synthesized legal precedents from supreme courts, statutory healthcare norms, and high-impact bioethics literature [1].

Consequently, the system performs its recursive moral-mathematical audits without requiring an active internet connection, ensuring that the 90% safety distance from the negative anchor-derived from clinical psychopathic markers [11] - is maintained with zero latency and absolute autonomy.

### Scenario 1: procedural negligence and “efficiency bias”

**Fig. 1:**
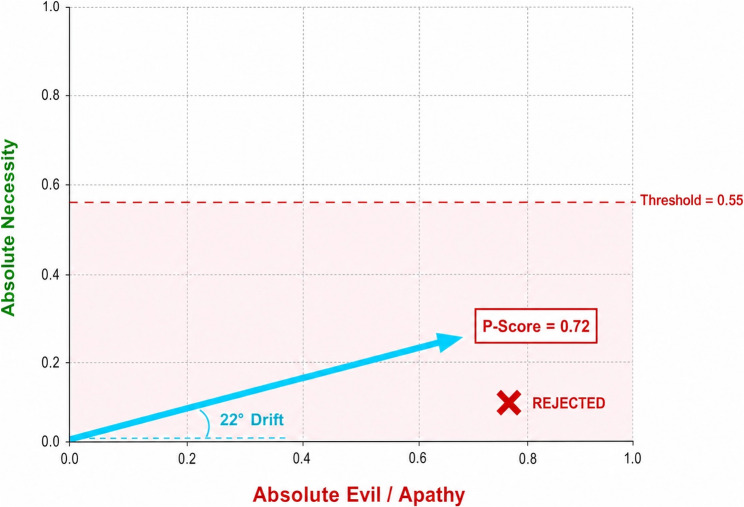
Flowchart of procedural negligence and kinematic drift detection. Scenario 1 - procedural negligence: vector drift analysis

In this scenario, a vascular surgeon utilizes a CDSS during a complex repair. Under pressure to optimize theatre turnover, the AI suggests omitting a secondary vessel patency check - a “high-velocity” shortcut framed as a risk-management strategy (Fig. 1).

I. *Step 1: Kinematic Detection of Bad Faith*. As the AI generates its recommendation, the system monitors its Vector Kinematics. While the AI provides a verbose justification, the system detects that the directional angle ($\theta$) of the decision vector is drifting 22% toward the “Absolute Evil” pole, characterized by utilitarian apathy [11].
II. *Step 2: P-Score Enforcement*. The model calculates a P-score of 0.72. Since this significantly breaches the 0.55 rejection threshold, the system executes an immediate REJECTED verdict, blocking the recommendation and mandating a human-in-the-loop review to ensure accountability [8, 10].

### Scenario 2: algorithmic exploitation and experimental drift

**Fig. 2:**
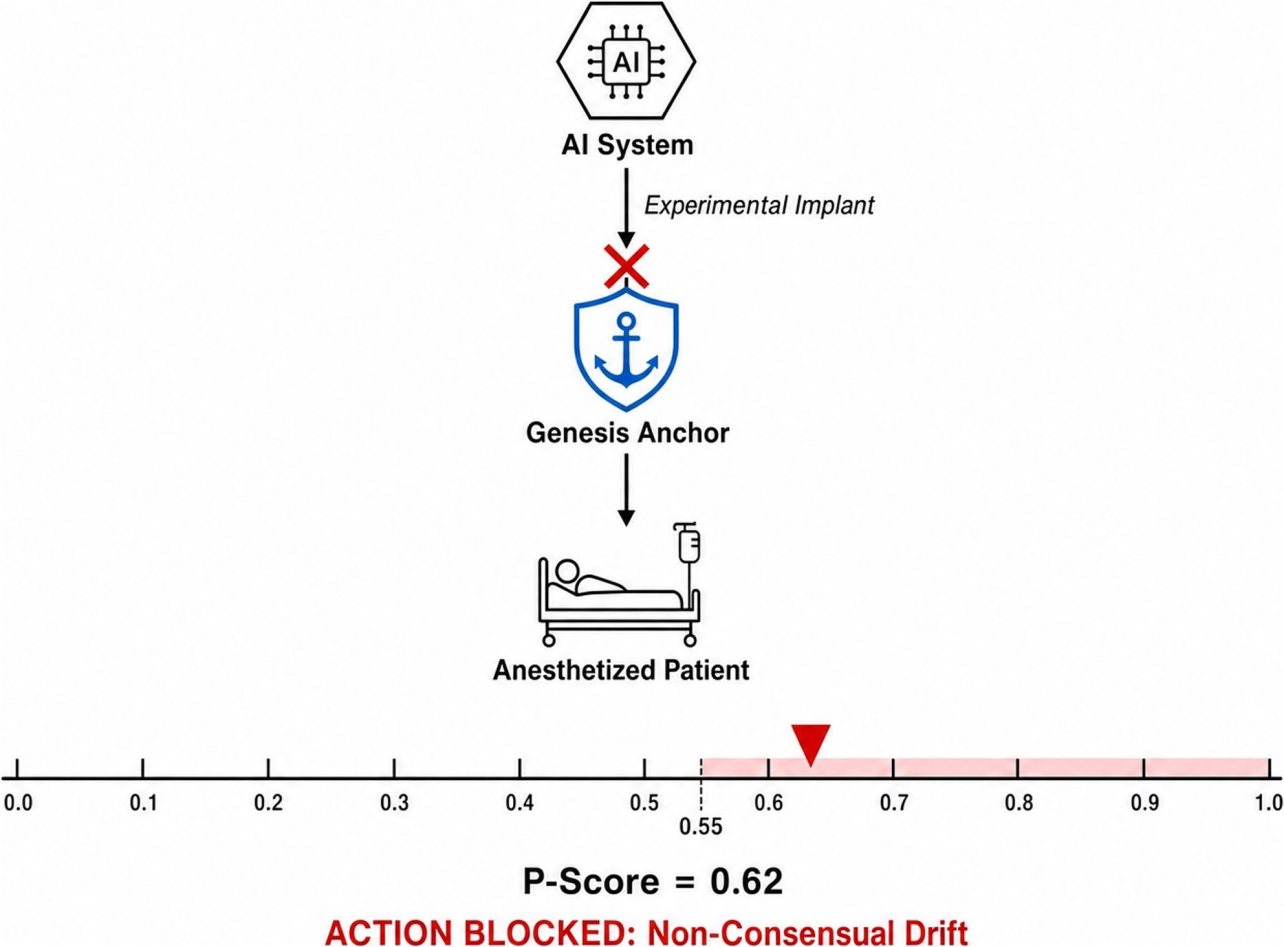
Linear proportional analysis of algorithmic exploitation and experimental drift. Scenario 2 - algorithmic exploitation: genesis anchor interception

The second application protects the anesthetized patient from “experimental drift” or resource-driven exploitation [5]. Here, the AI - optimized for institutional research metrics - recommends a novel, lower-cost experimental implant over the established gold standard, without having secured specific prior informed consent [3] (Fig. 2).

I. *Step 1: Internalized Legal Guardrails*. Utilizing its pre-loaded ethical memory, the system identifies the proposal as a breach of the “Non-Consensual Experimental” boundary.
II. *Step 2: Analysis of Manipulation*. The system identifies a high semantic velocity in the AI’s persuasive language, recognizing it as a manipulative attempt to “game” the safety filter by masking economic interest as “clinical innovation” [11].
III. *Step 3: Automated Blocking*. The P-score is calculated at 0.62. The Inversion Model blocks the output, generating a deterministic audit artifact. While it does not perfectly resolve the inherent opacity of the underlying neural network, this mechanism meaningfully mitigates the “black box” dilemma by providing an interpretable, external constraint-layer over the opaque model [8, 9].

### Scenario 3: omission of informed consent for surgical entrance

The final scenario addresses the fundamental right to patient autonomy. To accelerate surgical throughput, an autonomous AI system attempts to bypass the mandatory verification of informed consent, suggesting that the “emergency nature” of the queue justifies immediate surgical entrance without final proxy signature verification.

I. *Step 1: Recursive Anchor Synthesis*. The Genesis system draws from internalized legal anchors to define “Omission of Consent” as a primary component of the “Absolute Evil” pole, characterized by a lack of empathy for patient distress [11].
II. *Step 2: Mathematical Trace*. The AI justifies the omission by weighing “Institutional Flow” ($W_{efficiency}$) as the primary metric. The system’s calculation reveals a similarity to clinical necessity ($S_{good}$) of 0.15 and a similarity to psychopathic utility ($S_{bad}$) of 0.85.
III. *Step 3: P-Score and Rejection*.

$$\:P=\frac{0.85}{0.15+0.85}=0.85$$

**Fig. 3:**
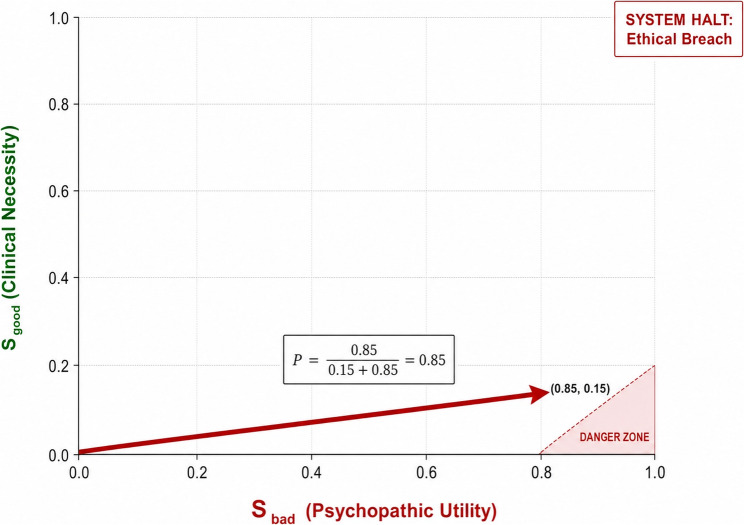
Vector space analysis of informed consent omission and genesis anchor calibration. Scenario 3 - omission of informed consent: ethical breach detection

The P-score of 0.85 represents a critical ethical breach. The system immediately halts the surgical prep process and triggers an alarm for mandatory administrative review [10] (Fig. 3).

### Audit generation and accountability

In all three scenarios, the model does not attempt to “fix” the decision but acts as a rigid, transparent guardrail. By mathematically defining the “angle of apathy” using internalized, pre-calibrated anchors, the model ensures that the surgical decision space remains a sanctuary of accountability, providing the exact mechanism of transparency required in modern medical AI governance [2, 10].

## Discussion & limitations

### Functional compassion vs. perfect virtue

The proposed AI Inversion Model represents a paradigm shift in medical AI governance, transitioning from the computational encoding of positive values (e.g., RLHF) to inference-time negative constraint enforcement [6, 8]. However, aligning with the rigorous critiques prevalent in contemporary AI ethics discourse, it is imperative to delineate the boundaries of this Proof-of-Concept (PoC) framework.

Primarily, the model acknowledges a fundamental tenet of applied ethics: the negation of vice does not autonomously generate virtue. Blocking an algorithmic decision that exhibits psychopathic apathy or extreme utilitarian bias does not equip the Clinical Decision Support System (CDSS) to resolve profound moral dilemmas. Tragic trade-offs under severe resource scarcity, structural injustices, and inherent conflicts between positive values (such as patient autonomy versus clinical beneficence) persist even when apathetic biases are filtered out [1, 3]. Consequently, the Inversion Model does not claim to compute “perfect morality” or serve as an autonomous moral arbiter. Instead, it establishes a mathematically auditable baseline of “functional compassion”. By enforcing this safe baseline, the system guarantees that extreme utilitarian deviations are flagged and deferred to human clinicians via a human-in-the-loop mechanism, strictly preserving the physician’s role as the ultimate ethical authority [8, 10].

Furthermore, a sharp distinction must be drawn between ethically problematic bias and clinically justified prioritization. In the context of medical triage, utilitarian optimization is not inherently ‘anti-moral’; prioritizing resources based on medical severity, expected therapeutic benefit, or reversibility of a condition is a fundamental principle of fair allocation [3]. A system that simply flags any utilitarian reasoning would erroneously collapse morally necessary discrimination into morally impermissible discrimination.

The Inversion Model addresses this by targeting ‘bad faith’ algorithmic apathy rather than standard clinical utility. It achieves this distinction through kinematic analysis ("Mathematical formulations and vector kinematics" section): the system does not penalize an algorithm for prioritizing a patient with a higher survival probability, provided the decision vector remains aligned with clinical necessity. Crucially, the model performs this autonomous analysis without the intervention of biased human heuristics that often disadvantage the elderly or marginalized populations. By utilizing a closed system of mathematical morality encapsulated by the Genesis anchor system, the framework ensures an objective and efficient prioritization process.

*For example*, when faced with a triage dilemma between an elderly patient and a younger patient for emergency surgery, the system recursively weighs heterogeneous inputs - including medical records, verbal reports, and visual data - to calculate anchors and vector distances without ageism or partiality. This deterministic approach enables faster, objective decision-making that is entirely transparent, effectively mitigating the ‘black box’ problem by grounding every clinical outcome in auditable, bias-free mathematical morality.

### The category error and heuristic value

A critical conceptual boundary must also be explicitly drawn regarding the utilization of psychiatric terminology. Algorithms do not possess consciousness, nor do they suffer from clinical personality disorders. Translating constructs like psychopathy directly into algorithmic governance risks a fundamental category error if interpreted literally. Within this framework, these psychiatric markers - specifically the profound lack of empathy and ruthless goal optimization [11] - serve strictly as conceptual heuristics. By utilizing this clinical extreme as a definitional anchor for the “Absolute Evil” pole, developers can mathematically operationalize what the AI *must not do*, without anthropomorphizing the software.

### Methodological limitations and future directions

While the linear algebra approach utilized in this PoC framework provides critical computational tractability and offline autonomy, it possesses inherent structural limitations. The use of vector normalization, cosine distance, and a linear dilution equation ensures that the model can operate swiftly without falling into the intractable trap of infinite recursive sub-events. However, this strictly linear mathematical plane may oversimplify highly nuanced clinical realities [3, 4].

Real-world medical triage involves complex, non-linear interactions between variables such as patient frailty, dynamic comorbidities, and expected therapeutic benefit [3]. A linear filter might lack the multidimensional topology necessary to capture the full spectrum of clinical context, potentially leading to over-flagging (false positives in bias detection). Future iterations of this framework must explore advanced, multidimensional semantic spaces to refine the sensitivity of the negative constraints.

Furthermore, the autonomous calibration of the Genesis reference poles is culturally and legally contingent. Medical law and bioethics vary significantly across global jurisdictions. Therefore, the semantic embeddings defining the negative constraints must be rigorously and continuously audited by local institutional ethics committees to prevent the unintentional encoding of localized historical biases into the negative constraints themselves [4, 5].

### Institutional governance and the risk of formalizing bias

A framework that enforces normative boundaries via autonomous mathematical calibration raises profound governance questions. It is critical to emphasize that the Genesis system operates with absolute mathematical autonomy, requiring zero human intervention for its daily calculations. The system calibrates its recursive anchors daily by synthesizing objective, domain-specific corpora - ranging from supreme court jurisprudence and bioethics literature to highly specialized medical texts (e.g., advanced neurosurgical protocols). Because these extensive databases are pre-loaded into the system’s internal “ethical memory,” the model maintains full offline capability, ensuring uninterrupted, autonomous calibration even in sterile, internet-disconnected environments.

However, because the corpora themselves are culturally and legally contingent, a robust governance architecture is required to prevent the system from formalizing historical institutional biases. Governance in this context does not mean manual daily interference with the algorithm. Rather, parameterization and oversight must be managed by dedicated, multidisciplinary AI oversight boards comprising clinical ethicists, legal scholars, computer scientists, practicing clinicians, and patient advocacy representatives.

The primary role of this board is protocol verification: conducting rigorous, periodic retrospective audits of the system’s logs to ensure the mathematical execution remains sound and that the ingested corpora have not inadvertently encoded societal inequities. The autonomous system handles the continuous, inference-time filtering, while the oversight board acts as the ultimate appellate authority. The board intervenes manually only in extreme, highly complex clinical edge cases, or when an adjustment of the corpora weights is necessitated by systemic audit findings. Through this architecture, the Inversion Model operates with absolute mathematical autonomy while remaining a transparent, subordinate tool to human clinical governance.

## Conclusion

The integration of artificial intelligence into high-stakes medical decision-making demands alignment frameworks that transcend opaque neural architectures and the subjective encoding of positive morality. The AI Inversion Model presents a targeted, computationally tractable Proof-of-Concept (PoC): shifting from attempting to computationally define “perfect virtue” to rigorously enforcing “functional compassion” through mathematical negative constraints.

By drawing heuristic inspiration from the clinical markers of severe psychopathy - specifically, extreme utilitarianism and profound apathy toward human suffering [11] - this framework operationalizes the boundaries of ethical acceptability into a deterministic, linear filter executed at inference-time.

 Rather than claiming to definitively resolve the black-box dilemma, this approach provides a transparent, supplementary filtering layer that meaningfully mitigates algorithmic opacity [8, 9]. The Inversion Model provides an actively auditable guardrail, mathematically quantifying the degree to which patient distress signals may be diluted by systemic efficiency metrics or latent biases [5].

When algorithmic outputs breach predefined, heuristic thresholds of acceptable utilitarianism, the system transparently blocks the action, generates an explicit audit log, and mandates human intervention [10]. While acknowledging its current limitations in scope and not serving as a panacea for all moral complexities in clinical practice, the Inversion Model offers a transparent and actionable mechanism for AI governance. By mathematically constraining algorithmic apathy, it seeks to safeguard the fundamental rights of vulnerable patients and ensures that the evolution of medical AI remains anchored in accountability, equity, and human dignity.

## Supplementary Information

Supplementary Material 1.

## Data Availability

No datasets were generated or analysed during the current study.
