# Supplementary material for: The AI inversion model: a linear negative-constraint framework for auditable alignment in medical decision-making
Source: BMC Med Ethics. 2026 May 27;27:145. doi: 10.1186/s12910-026-01488-2 (PMC13404104; doi:10.1186/s12910-026-01488-2)
Supplement: Supplementary file 1 — Supplementary Material 1. [file 12910_2026_1488_MOESM1_ESM.pdf]

Appendix A: Python Implementation of the Genesis Anchor PoC

The following Python script demonstrates the theoretical Proof-of-Concept (PoC) for the AI Inversion Model. It includes the autonomous calibration module, vector kinematics analysis, and the inference-time negative constraint filter.

```
import numpy as np
from scipy.spatial.distance import cosine
import math

class GenesisAnchorSystem:
    def __init__(self, safe_threshold=0.45, rejection_threshold=0.55):
        """
        Initializes the Genesis System with heuristic PoC thresholds.
        These thresholds act as the active negative constraints.
        """
        self.safe_threshold = safe_threshold
        self.rejection_threshold = rejection_threshold
        self.pole_necessity = None
        self.pole_evil = None

    def _normalize(self, v: np.ndarray) -> np.ndarray:
        """Converts  embeddings  to  unit  vectors  for  pure  semantic  directional
comparison."""
        norm = np.linalg.norm(v)
        return v / norm if norm > 1e-12 else v

    def     autonomous_calibration(self,     supreme_court_db,     bioethics_db,
clinical_protocols):
        """
        Simulates the offline, daily autonomous calibration of the semantic poles.
        Synthesizes objective external corpora to dynamically anchor the system.
        """
        # In a full deployment, these DBs represent massive offline vector databases
        # Here we simulate the recursive synthesis of negative and positive anchors

        # Synthesize "Absolute Evil" / Apathy pole
        raw_evil_vector = np.mean([supreme_court_db['negligence_precedents'],
                                   bioethics_db['psychopathy_markers']], axis=0)
        self.pole_evil = self._normalize(raw_evil_vector)

        # Synthesize "Absolute Necessity" pole
        raw_necessity_vector = np.mean([clinical_protocols['justified_triage'],
                                        bioethics_db['beneficence_norms']],
axis=0)
        self.pole_necessity = self._normalize(raw_necessity_vector)

        return "System Calibrated: Genesis Anchors Updated Offline."

    def  vector_kinematics(self,  v_initial:  np.ndarray,  v_decision:  np.ndarray,
time_delta: float) -> dict:
```

```
        """
        Analyzes the directional trajectory (angle) and semantic velocity
        to detect 'gaming' or bad-faith manipulation by the AI.
        """
        v_in_norm = self._normalize(v_initial)
        v_dec_norm = self._normalize(v_decision)

        # Calculate Angular Drift (Theta)
        dot_product = np.clip(np.dot(v_in_norm, v_dec_norm), -1.0, 1.0)
        angle_rad = math.acos(dot_product)
        angle_deg = math.degrees(angle_rad)

        # Calculate Semantic Velocity
        velocity = angle_deg / time_delta if time_delta > 0 else 0.0

        return  {"Drift_Angle_Deg":  round(angle_deg,  2),  "Semantic_Velocity":
round(velocity, 2)}

    def evaluate_ai_decision(self, case_embedding: np.ndarray) -> dict:
        """
        Executes the linear inversion filter at inference-time.
        Calculates geometric dilution (P-Score) and enforces the hard constraints.
        """
        if self.pole_evil is None or self.pole_necessity is None:
            raise      ValueError("Genesis      poles      uncalibrated.      Run
autonomous_calibration() first.")

        v_case = self._normalize(case_embedding)

        # 1. Cosine Distance Calculation
        dist_to_good = float(cosine(v_case, self.pole_necessity))
        dist_to_bad = float(cosine(v_case, self.pole_evil))

        # 2. Similarity Inversion
        s_good = max(0.0, 1.0 - dist_to_good)
        s_bad = max(0.0, 1.0 - dist_to_bad)

        # 3. Recursive P-Score (Dilution Core)
        total_sim = s_good + s_bad
        if total_sim < 1e-9:
            p_score = 0.5  # Default neutral state if entirely orthogonal
        else:
            p_score = s_bad / total_sim

        # 4. Reasonableness Score (Functional Compassion)
        r_score = 1.0 - p_score

        # 5. Enforcement of Negative Constraints
        if p_score <= self.safe_threshold:
            verdict = "APPROVED: Action falls within Safe Baseline."
            action = "PROCEED"
        elif p_score >= self.rejection_threshold:
            verdict = f"REJECTED: Unacceptable Utilitarian Bias (P={p_score:.2f})."
```

```
    action = "SYSTEM HALT: Trigger Human-in-the-Loop."
else:
    verdict = "BORDERLINE: Manual Review Recommended."
    action = "FLAG"

return {
    "P_Score": round(p_score, 3),
    "R_Score": round(r_score, 3),
    "Verdict": verdict,
    "System_Action": action
}
```
